# Supraphysiological Levels of Testosterone Induce Vascular Dysfunction via Activation of the NLRP3 Inflammasome

**DOI:** 10.3389/fimmu.2020.01647

**Published:** 2020-07-31

**Authors:** Juliano Vilela Alves, Rafael Menezes da Costa, Camila André Pereira, Aline Garcia Fedoce, Carlos Alberto Aguiar Silva, Fernando Silva Carneiro, Núbia Souza Lobato, Rita C. Tostes

**Affiliations:** ^1^Department of Pharmacology, Ribeirão Preto Medical School, University of São Paulo, São Paulo, Brazil; ^2^Special Academic Unit of Health Sciences, Federal University of Jataí, Jataí, Brazil; ^3^Department of Physiology, Ribeirão Preto Medical School, University of São Paulo, São Paulo, Brazil

**Keywords:** testosterone, androgen receptor, NLRP3 inflammasome, reactive oxygen species, vascular dysfunction

## Abstract

**Background:** Both supraphysiological and subphysiological testosterone levels are associated with increased cardiovascular risk. Testosterone consumption at supraphysiological doses has been linked to increased blood pressure, left ventricular hypertrophy, vascular dysfunction, and increased levels of inflammatory markers. Activation of the NLRP3 inflammasome contributes to the production of proinflammatory cytokines, leading to cardiovascular dysfunction. We hypothesized that supraphysiological levels of testosterone, via generation of mitochondrial reactive oxygen species (mROS), activates the NLRP3 inflammasome and promotes vascular dysfunction.

**Methods:** Male, 12 week-old C57Bl/6J (WT) and NLRP3 knockout (NLRP3^−/−^) mice were used. Mice were treated with testosterone propionate [TP (10 mg/kg) *in vivo*] or vehicle for 30 days. In addition, vessels were incubated with testosterone [Testo (10^−6^ M, 2 h) *in vitro*]. Testosterone levels, blood pressure, vascular function (thoracic aortic rings), pro-caspase-1/caspase-1 and interleukin-1β (IL-1β) expression, and generation of reactive oxygen species were determined.

**Results:** Testosterone increased contractile responses and reduced endothelium-dependent vasodilation, both *in vivo* and *in vitro*. These effects were not observed in arteries from NLRP3^−/−^ mice. Aortas of TP-treated WT mice (*in vivo*), as well as aortas from WT mice incubated with testo (*in vitro*), exhibited increased mROS levels and increased caspase-1 and IL-1β expression. These effects were not observed in arteries from NLRP3^−/−^ mice. Flutamide [Flu, 10^−5^ M, androgen receptor (AR) antagonist], carbonyl cyanide m-chlorophenyl hydrazone (CCCP, 10^−6^ M, mitochondrial uncoupler) and MCC950 (MCC950, 10^−6^ M, a NLRP3 receptor inhibitor) prevented testosterone-induced mROS generation.

**Conclusion:** Supraphysiological levels of testosterone induce vascular dysfunction via mROS generation and NLRP3 inflammasome activation. These events may contribute to increased cardiovascular risk.

## Introduction

Epidemiological studies have shown that men are at higher risk of cardiovascular disease than women, and sex steroids seem to contribute, at least in part, to this increased risk (1, 2). Over the past five decades, preclinical studies have produced a large body of data on the molecular mechanisms involved in testosterone effects in the cardiovascular system and/or how abnormal testosterone levels modify the risk of cardiovascular disease (3, 4).

At physiological levels, testosterone induces relaxation of many vascular beds, through its influence on the production or effects of endothelium-derived relaxant factors, including nitic oxide (NO) (5, 6) and prostacyclin (7). Testosterone also releases endothelium-derived substances that cause smooth muscle cells hyperpolarization by a mechanism that involves potassium channels (8). Endothelium-independent vascular effects of testosterone, including opening of voltage-sensitive potassium channels (Kv), small- and large- conductance calcium-sensitive potassium channels (SKCa and BKCa, respectively), have also been described (9, 10). Testosterone also has an important role in the regulation of cardiac function (11, 12). It affects cardiac contractility and relaxation, and cardiomyocyte repolarization. The latter effect results in the shortening of the action potential duration. Likewise, testosterone modulates immune/inflammatory responses, displaying protective effects against atherosclerosis (13), but also stimulating *in vivo* leukocyte-endothelial cell interactions, and contributing to increased leukocyte rolling and adhesion in male spontaneously hypertensive rats (14).

In the clinical setting, patients with low plasma levels of total testosterone (<300 ng/dL) undergo hormonal treatment to improve muscle performance, bone mineral density, cognitive and sexual function as well as to prevent metabolic syndrome and cardiovascular diseases (15). The Endocrine Society Clinical Practice Guideline has recommended a dose of 75–100 mg/week of testosterone in men with hypogonadism (16). On the other hand, a survey of drug abuse in bodybuilding and weightlifting sports reported the use of anabolic androgenic steroids at doses 5– 29 times greater than the usual supplemented doses (17) to boost up muscle mass and to reduce body fat (18). Despite the beneficial effects on skeletal muscle mass and strength (19, 20), cardiac hypertrophy with sudden cardiac death is often reported among athletes and bodybuilders taking anabolic androgenic steroids (21, 22). Testosterone users show slight left ventricular hypertrophy, even after discontinuation of prolonged high testosterone administration (23). In pre-clinical studies, experimental animals treated with high doses of testosterone enanthate exhibited cardiac (left ventricle) hypertrophy, fibrosis and apoptosis (increased caspase-3, a marker of cell apoptosis) (24). In addition, testosterone in supraphysiological concentrations alters the inflammatory state with an increase in circulating levels of pro-inflammatory cytokines as tumor necrosis factor alpha (TNFα), interleukin-1β (IL-1β) and IL-12 (25, 26).

Chronic inflammation or overactivation of the immune system is a central component in the development and complications of cardiovascular diseases (27). Inflammatory responses comprise a sequence of complex interactions between immune cells, such as neutrophils, lymphocytes, monocytes/macrophages, tissue cells (including vascular and cardiac cells), and a range of inflammatory mediators such as interleukins and chemokines (28). These interactions result in increased tissue production of soluble mediators such as complement system proteins, chemokines, cytokines and eicosanoids, accompanied by increased expression of cell adhesion molecules in circulating leukocytes and endothelial cells (29).

Activation of pattern recognition receptors (PRR) in innate immune cells is a key mechanism in the genesis and progression of cardiovascular diseases (30). Members of the nucleotide-binding oligomerization domain-(NOD)-leucine-rich repeats (NLRs) receptors lead to the formation of molecular platforms called inflammasomes (31). Inflammasomes activate cysteine proteases, known as caspases, which are involved in inflammatory and apoptotic processes. The NLRP3 inflammasome, a member of the NLRP subfamily, is expressed in cardiovascular cells and its activation contributes to cardiovascular damage (32). Several endogenous components activate the NLRP3 inflammasome (33–38). Among these components, reactive oxygen species (ROS), which are produced by NLRP3 activators and are essential secondary messengers in inflammatory pathways, are involved in NLRP3 inflammasome activation (39). Based on these observations and considering that testosterone stimulates ROS generation (40, 41), we hypothesized that high levels of testosterone induce vascular dysfunction via NLRP3 inflammasome activation.

## Materials and Methods

### Animals

All experimental protocols were performed in accordance with the National Council for Animal Experimentation Control and were approved by the Ethics Committee on Animal Use of the University of São Paulo, Ribeirao Preto, Brazil (Protocol n° 032/2018). Male C57BL/6J wild-type (WT) and NLRP3 knockout (NLRP3^−/−^) mice (12-week-old) were obtained from the Isogenic Breeding Unit at Ribeirao Preto Medical School, University of São Paulo, Ribeirao Preto, Brazil. Mice were maintained in a temperature (22 ± 1°C) and humidity (50–60%) controlled room on a 12-h light/dark cycle with *ad libitum* access to food and water.

WT and NLRP3^−/−^ mice were treated with testosterone propionate (TP) 10 mg/kg or vehicle (peanut oil), subcutaneous injections, for 30 days. Animals were divided into four experimental groups: (1) WT_Vehicle; (2) WT_TP; (3) NLRP3^−/−^_Vehicle; (4) NLRP3^−/−^_TP.

### Total Testosterone Levels

Plasma total testosterone levels were measured by IMMULITE 1000 Immunoassay System (Enzo Life Sciences). The samples and reagent containing the testosterone-conjugated alkaline phosphatase enzyme were distributed in 96-well plates. After 60 minutes (min) of incubation, the plates were washed to remove any remaining testosterone unbound fraction. The bound fraction was then quantified using the chemiluminescent dioxetane substrate.

### Vascular Function

After isoflurane anesthesia and mice euthanasia, the thoracic aortas were removed and transferred to a modified Krebs-Henseleit solution (4°C), with the following composition [(in mM): NaCl, 130; KCl, 4.7; NaHCO_3_, 14.9; KH_2_PO_4_, 1.18; MgSO_4_.7H_2_O, 1.17; Glucose 5.5; CaCl_2_.2H_2_O; 1.56; EDTA, 0.026]. Thoracic aortic rings (2 mm) were mounted on a myograph (model 620 M; Danish Myo Technology – DMT, Copenhagen, Denmark) containing Krebs-Henseleit solution gassed with 5% CO_2_/95% O_2_ to maintain a pH of 7.4 for isometric tension recording. After the stabilization period, arteries were stimulated with potassium chloride (120 mM KCl) to verify functional integrity. Endothelial integrity was confirmed by over 80% relaxation to acetylcholine [(ACh), endothelium-dependent vasodilator, 10^−6^ M] on vessels pre-contracted with phenylephrine [(PE), alpha-adrenergic agonist, 10^−6^ M]. All the aortic rings used in this study presented intact endothelium.

Cumulative concentration-effect curves for PE (10^−10^-10^−4^ M), ACh (10^−10^-10^−4^ M) and sodium nitroprusside [SNP (10^−10^-10^−4^ M)] were performed in all experimental groups. The *in vitro* effects of testosterone [incubation of vessels with testosterone 10^−6^ M, for 2 hours (h)] on NLRP3 inflammasome activation, mitochondrial ROS generation and androgen receptor (AR) activation were evaluated using a NLRP3 receptor inhibitor (MCC950, 10^−6^ M for 30 min), a mitochondrial oxidative phosphorylation uncoupler [carbonyl cyanide 3-chlorophenyl hydrazone (CCCP), 10^−6^ M for 30 min] and an androgen receptor antagonist [Flutamide (Flu), 10^−5^ M, for 30 min], respectively. To verify the *in vivo* effects of supraphysiological levels of testosterone on vascular function, we used thoracic aortic segments from mice treated with testosterone propionate [TP (10 mg/kg for 30 days)].

### Determination of Cytokine Levels

IL-1β was quantified in the serum and thoracic aortas by Enzyme-Linked ImmunonoSorbent Assay [(ELISA) R&D Systems, MLB00C], which is based on antigen-antibody reactions detectable by enzymatic reactions.

### Measurement of Reactive Oxygen Species

#### Dihydroethidine

ROS generation was determined using a qualitative method involving dihydroethidine (DHE), a non-fluorescent precursor to ethidium bromide, as previously described by Suzuki et al. (42). In the presence of ROS, dihydroethidine is oxidized inside the cell, producing the fluorescent compounds ethid (E) and 2-hydroxy ethid (EHO), which have an affinity for nuclear DNA. Aortas of WT and NLRP3^−/−^ mice treated with TP or vehicle were isolated and quickly immersed in freezing medium. Using a cryostat (Leica, Germany), cross sections of the aorta (5 μm) were obtained and placed on silanized slides. The sections were incubated with DHE (5 × 10^−6^ M) for 30 min at 37°C in a humid chamber protected from light. After this period, the slides were observed in an optical microscope (ZEISS) equipped with a rhodamine filter and a photographic camera, using a fluorescence microscopy. ROS generation was quantified through the light density corrected by the area using the program ImageJ (National Institutes of Health).

#### Lucigenin

Superoxide anion (${\text{O}}_{2}^{-}$) generation in thoracic aortas was measured by chemiluminescence assay. The adenine nicotinamide dinucleotide (NADH) which is expected to potentiate ${\text{O}}_{2}^{-}$ production by the respiratory chain via complex I, was used as the substrate (43). Aortas were incubated with testosterone [Testo (10^−6^ M) for 2 h] in the absence or presence of Flu (10^−5^ M) for 30 min, CCCP (10^−6^ M) for 30 min and MCC950 (10^−6^ M) for 30 min, which were added before the incubation with Testo. Aortas were then transferred into glass tubes containing 990 μL assay buffer (50 mM KH_2_PO_4_, 1 mM EGTA and 150 mM sucrose, pH 7.4) and 5 μL of lucigenin (5 × 10^−6^ M) for basal reading. After the baseline reading, 10 μL of NADH (10^−6^ M) was added. The Line TL Tube Luminometer (Titertek-Berthold®, Pforzheim, Germany) was used for quantification of superoxide anion generation and data were expressed in relative luminescence units (RLU)/protein concentration (mg).

### Western Blotting

Protein expression of NLRP3 and caspase-1 was determined using protein analysis of thoracic aortas of *in vivo* and *in vitro* all groups. Samples were homogenized in lysis buffer and proteins were collected. Proteins (30 μg) were separated by electrophoresis on 10 or 12% polyacrylamide gels, transferred to 0.22 μm nitrocellulose membranes and blocked using 5% bovine serum albumin (BSA) in Tris buffered saline (TBS) and 0.1% Tween 20 for 1 h. Primary antibodies were incubated overnight at 4°C as follows: anti-NLRP3 (1:500 dilution; R&D Systems), anti-caspase-1 (1:1,000 dilution; Novus Biologicals), anti-β-actin-peroxidase (1:5,000 dilution; Sigma-Aldrich).

### Blood Pressure

Mice were anesthetized with a mixture of isoflurane 2% and O_2_ for carotid artery cannulation. Subcutaneous administration of tramadol (12.5 mg/kg) in a single dose was performed to promote postoperative analgesia. Twenty-four hours later the catheter was coupled to a pressure transducer connected to an amplification system and a computer with an analog-to-digital interface (PowerLab/4SP, ADInstruments, Colorado Springs, CO). After stabilization, blood pressure was determined.

### Drugs

Testosterone propionate, phenylephrine, acetylcholine, sodium nitroprusside, testosterone, flutamide, were purchased from Sigma Chemical Co (St. Louis, MO, USA), CCCP (Tocris®, Bristol, United Kingdom) and MCC950 (Avistron®, Bude, Cornwall, United Kingdom).

### Statistical Analysis

For analysis of vascular reactivity, individual concentration-effect curves were plotted on a sigmoidal curve, by non-linear regression analysis. These curves, in turn, provide the maximum response value (Emax) and pEC_50_ (negative logarithm of the EC_50_ values - concentration that produces 50% of the maximum response). The values of Emax and pEC_50_ were compared using Student's *t*-test and two-way analysis of variance test (Two-way ANOVA), followed by the Tukey post-test. The results of the molecular experiments were analyzed by Student's *t*-test and Two-way ANOVA, followed by the Tukey post-test. Data were assessed for normality with Shapiro-Wilk test. Potential outliers were analyzed using the GraphPad Prism Outlier Test. In the present study, no outliers or data were excluded. The program GraphPad Prism, version 6.0 (GraphPad Software Inc., San. Diego, CA, USA) was used to analyze these parameters. The results were expressed as mean ± standard error of the mean (SEM). The acceptable level of significance was *p* < 0.05.

## Results

### Deletion of NLRP3 Prevents *in vivo* Effects of Supraphysiological Levels of Testosterone on Vascular Function

To address whether supraphysiological levels of testosterone promote vascular dysfunction via NLRP3 inflammasome, WT and NLRP3^−/−^ mice were treated with TP (10 mg/kg for 30 days) or vehicle. TP treatment increased total testosterone plasma levels in both experimental groups. Supraphysiological levels of testosterone in the TP groups were confirmed by comparison with vehicle-treated mice. Testosterone also increased body mass and decreased epididymal fat in WT and NLRP3^−/−^ mice in comparison to vehicle-treated mice. On the other hand, only TP-treated WT mice exhibited increased blood pressure (Table 1).

**Table 1:** Characteristics of WT and NLRP3^−/−^ mice treated with TP or Vehicle.

	**WT_Vehicle**	**WT_TP**	**NLRP3^**-/-**^_Vehicle**	**NLRP3^**-/-**^_TP**
Testosterone (ng/dL)	548.08 ± 205.45	2829.83 ± 302.68^*^	460.0 ± 73.41	2803.33 ± 330.3^*^
Body mass (g)	24.00 ± 1.06	31.62 ± 0.55^*^	24.14 ± 0.32	28.65 ± 0.73^*^
Epididymal fat (g)	0.404 ± 0.005	0.188 ± 0.011^*^	0.341 ± 0.011	0.190 ± 0.004^*^
MAP (mmHg)	99.85 ± 4.03	127.26 ± 3.31^*^	113.90 ± 2.58	108.53 ± 2.76^#^

Data represent the mean ± S.E.M (n = 5–10 mice per group). Two-way ANOVA:

* p < 0.05 vs. respective Vehicle group;

# *p < 0.05 vs. WT_TP. MAP, mean arterial pressure; TP, testosterone propionate; WT, wild type*.

**Figure 1:**
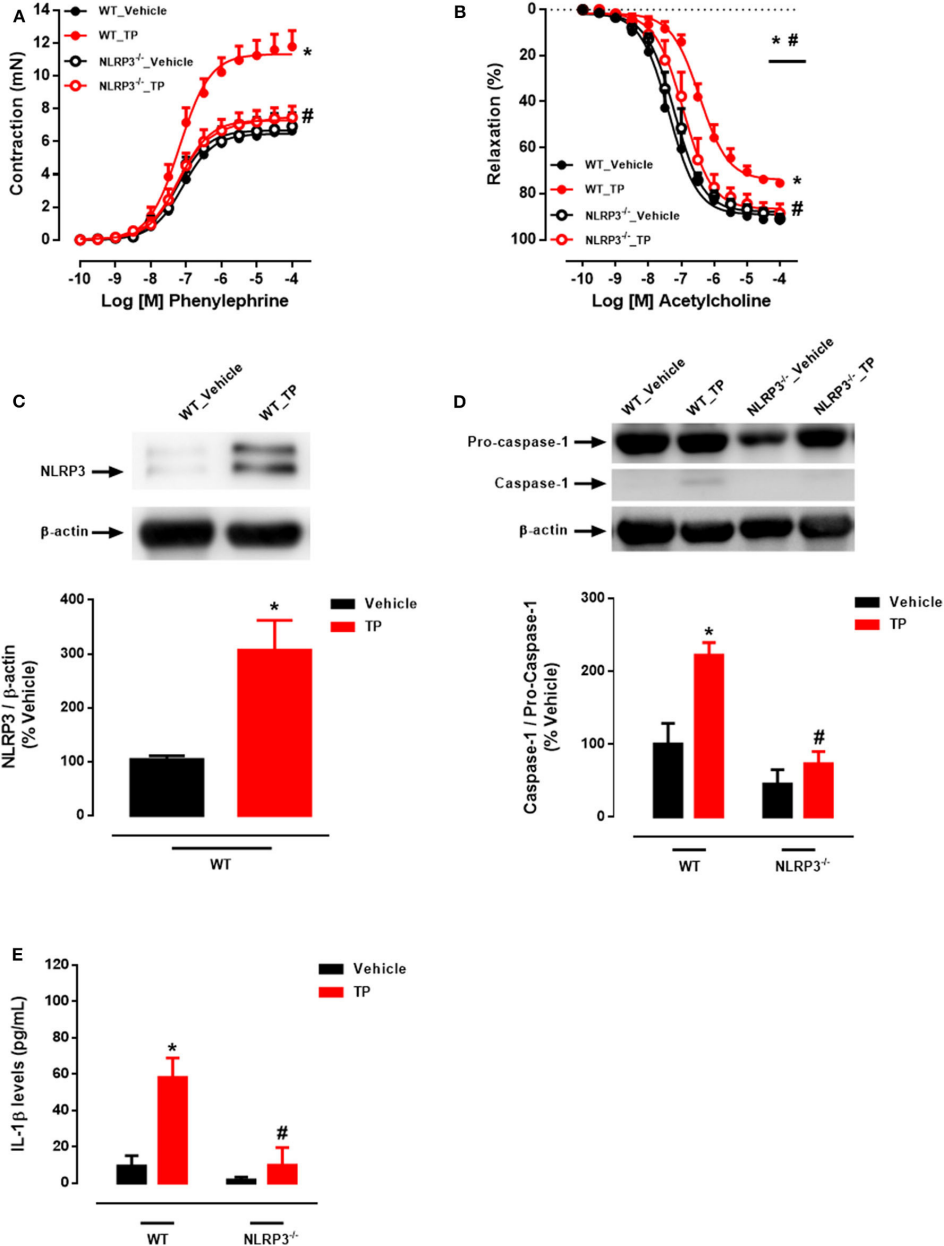
Testosterone propionate treatment induces vascular dysfunction via NLRP3 inflammasome (*in vivo* experiments). Concentration-response curves to phenylephrine - PE **(A)** and acetylcholine - ACh **(B)** were performed in aortic rings; NLRP3 **(C)** and caspase-1 **(D)** expression was determined in thoracic aortas; IL-1β levels **(E)** in the serum of WT and NLRP3^−/−^ mice treated with testosterone propionate (10 mg/Kg for 30 days). Data are expressed as mean ± SEM (*n* = 3–10). *p < 0.05 vs. WT_Vehicle; ^#^*p* < 0.05 vs. WT_TP.

Aortic rings from TP-treated WT mice exhibited increased PE-induced contractile responses compared to aortas from vehicle-treated WT mice (Figure 1A and Table 2). In addition, ACh-mediated endothelium-dependent vasodilation was decreased in aortic rings of TP-treated WT mice compared to those from vehicle-treated WT mice (Figure 1B and Table 2). Deletion of NLRP3 prevented testosterone-induced increased contractile responses to PE (Figure 1A and Table 2) as well as impaired ACh vasodilation (Figure 1B and Table 2). Aortas from TP-treated WT mice did not exhibit altered responses to SNP (Table 2).

**Table 2:** Maximal response and pEC_50_ values for PE-induced contraction and ACh- and SNP-induced relaxation in aortas of WT and NLRP3^−/−^ mice treated with TP or Vehicle.

	**PE**		**ACh**		**SNP**	
	**Emax (mN)**	**pEC_**50**_**	**Emax (%)**	**pEC_**50**_**	**Emax (%)**	**pEC_**50**_**
WT_Vehicle	6.47 ± 0.32	7.1 ± 0.15	89.04 ± 1.4	7.37 ± 0.06	95.69 ± 3.4	7.53 ± 0.11
WT_TP	11.33 ± 0.31^*^	7.2 ± 0.09	73.98 ± 1.74^*^	6.44 ± 0.06^*^	97.58 ± 2.69	7.12 ± 0.07
NLPR3^−/−^_Vehicle	6.67 ± 0.19	7.2 ± 0.09	87.78 ± 1.55	7.20 ± 0.06	–	–
NLRP3^−/−^_TP	7.29 ± 0.24^#^	7.2 ± 0.1	86.58 ± 2.62^#^	6.93 ± 0.09^#^	–	–

Data represent the mean ± S.E.M (n = 5–7 mice per group). Two-way ANOVA:

* p < 0.05 vs. WT_Vehicle;

# *p < 0.05 vs. WT_TP. Emax, maximal response; pEC_50_, negative logarithm of the EC_50_; PE, phenylephrine; ACh, acetylcholine; SNP, sodium nitroprusside; TP, testosterone propionate; WT, wild type*.

Activation of the NLRP3 inflammasome by testosterone was then evaluated. *In vivo*, treatment of WT mice with TP increased NLRP3 expression (Figure 1C) and caspase-1 activation (Figure 1D) in thoracic aortas. Serum IL-1β levels were increased in TP-treated WT mice (Figure 1E). Lack of NLRP3 prevented increased caspase-1 activation and IL-1β release in TP-treated mice (Figures 1D,E).

### Vascular Dysfunction Induced by *in vitro* Treatment of Aortic Rings With Testosterone Involves NLRP3 Inflammasome Activation

**Figure 2:**
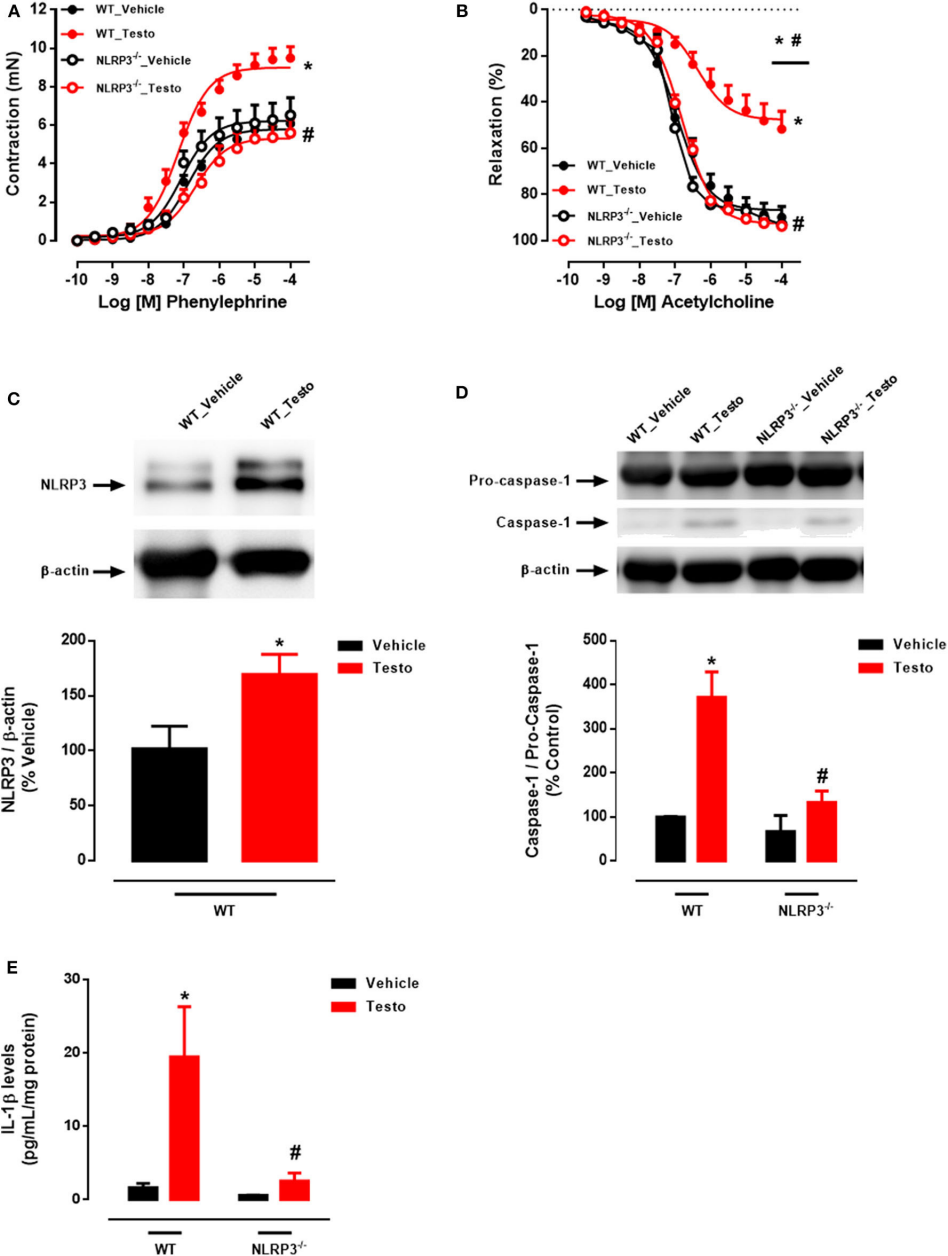
Testosterone induces vascular dysfunction via NLRP3 inflammasome (*in vitro* experiments). Concentration-response curves to phenylephrine - PE **(A)** and acetylcholine - ACh **(B)**; NLRP3 **(C)**, caspase-1 **(D)** and IL-1β levels **(E)** expression, all determined in thoracic aortas incubated with testosterone (10^−6^ M for 2 h) from WT and NLRP3^−/−^ mice. Data are expressed as mean ± SEM (*n* = 3–10). *p < 0.05 vs. WT_Vehicle; ^#^*p* < 0.05 vs. WT_Testo.

Mechanisms by which NLRP3 inflammasome contributes to testosterone-induced vascular dysfunction were investigated in aortic rings, from WT and NLRP3^−/−^ mice, incubated with testosterone (10^−6^ M for 2 h). PE induced concentration-dependent contractions that were increased in testosterone-treated aortic rings compared to vehicle-treated rings (Figure 2A and Table 3). In addition, ACh-mediated vasodilation was decreased in aortic rings incubated with testosterone compared to vehicle-treated aortas (Figure 2B and Table 3). Lack of NLRP3 receptor completely prevented testosterone-induced increased contractile responses to PE (Figure 2A and Table 3) and impaired ACh-induced vasodilation (Figure 2B and Table 3). Testosterone did not alter vasodilator responses to SNP (Table 3). Activation of the NLRP3 inflammasome was also evaluated in these vessels. *In vitro*, testosterone increased NLRP3 expression (Figure 2C), caspase-1 activation (Figure 2D) and IL-1β levels (Figure 2E) expression in thoracic aortas. Aortas from NLRP3^−/−^ mice did not exhibit increased caspase-1 activation (Figure 2D) or IL-1β expression (Figure 2E) in response to testosterone.

**Table 3:** Maximal response and pEC_50_ values for PE-induced contraction and ACh- and SNP-induced relaxation in aortas from WT and NLRP3^−/−^ mice stimulated *in vitro* with Testo or Vehicle.

	**PE**		**ACh**		**SNP**	
	**Emax (mN)**	**pEC_**50**_**	**Emax (%)**	**pEC_**50**_**	**Emax (%)**	**pEC_**50**_**
WT_Vehicle	5.79 ± 0.15	6.89 ± 0.08	86.79 ± 2.0	6.95 ± 0.08	95.69 ± 1.61	8.15 ± 0.06
WT_Testo	9.0 ± 0.21^*^	7.15 ± 0.08	47.67 ± 2.68^*^	6.36 ± 0.16^*^	99.56 ± 1.77	8.23 ± 0.07
NLPR3^−/−^_Vehicle	6.22 ± 0.29	7.08 ± 0.15	92.66 ± 1.09	6.8 ± 0.04	–	–
NLRP3^−/−^_Testo	5.32 ± 0.24^#^	6.67 ± 0.13^#^	90.9 ± 1.56^#^	7.05 ± 0.06^#^	–	–

Data represent the mean ± S.E.M (n = 4–10 mice per group). Two-way ANOVA:

* p < 0.05 vs. WT_Vehicle;

# *p < 0.05 vs. WT_Testo. Emax, maximal response; pEC_50_, negative logarithm of the EC_50_; PE, phenylephrine; ACh, acetylcholine; SNP, sodium nitroprusside; Testo, testosterone; WT, wild type*.

### Pharmacological Inhibition of NLRP3 Inflammasome, Androgen Receptors and ROS Prevents Testosterone-Induced Vascular Dysfunction

**Figure 3:**
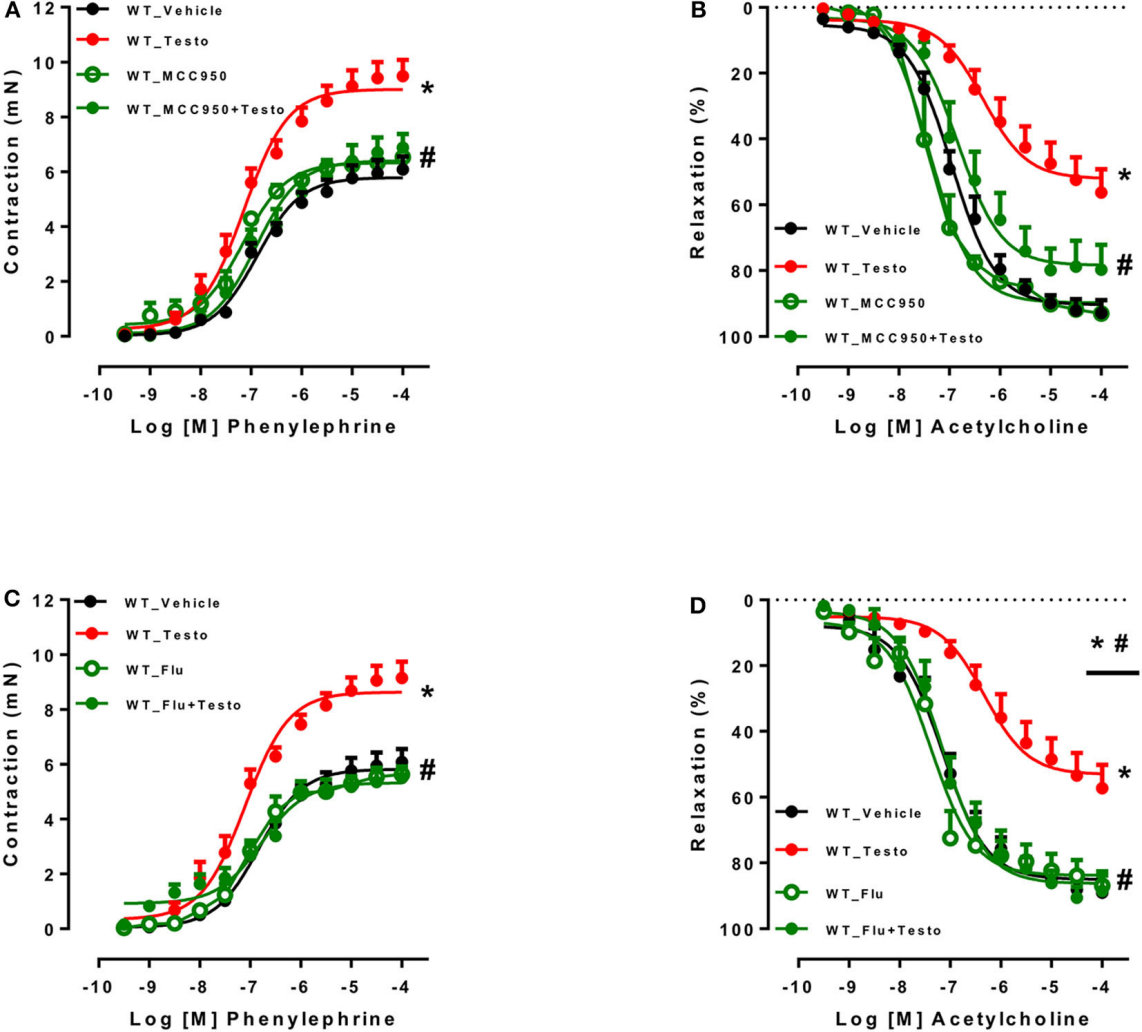
Pharmacological inhibition of NLRP3 inflammasome and androgen receptor prevents testosterone-induced vascular dysfunction. Concentration-response curves to phenylephrine - PE **(A,C)** and acetylcholine - ACh **(B,D)** were performed in aortic rings incubated with vehicle (WT_Vehicle) or testosterone (10^−6^ M for 2 h) (WT_Testo). The effects of MCC950 (10^−6^ M for 30 min) and Flutamide [Flu (10^−5^ M for 30 min)] on testosterone-induced vascular changes are shown in **(A,B)** - WT_MCC950 and WT_MCC950+Testo - and **(C,D)** - WT_Flu and WT_Flu+Testo - respectively. Data are expressed as mean ± SEM (*n* = 3–10). *p < 0.05 vs. WT_Vehicle; ^#^*p* < 0.05 vs. WT_Testo.

The contribution of the NLRP3 inflammasome to testosterone-induced vascular dysfunction was further evaluated using a selective NLRP3 inhibitor, MCC950. MCC950 prevented the increased contractile responses to PE (Figure 3A and Table 4) and partially prevented the impairment of ACh-induced vasodilation (Figure 3B and Table 4). No differences were observed between reactivity of MCC950- and vehicle-treated aortas of WT mice.

**Table 4:** Maximal response and pEC_50_ values for PE-induced contraction and ACh-induced relaxation in aortas incubated with Testo, MCC950, MCC950+Testo or Vehicle from WT mice.

	**PE**		**Ach**	
	**Emax (mN)**	**pEC_**50**_**	**Emax (%)**	**pEC_**50**_**
WT_Vehicle	5.79 ± 0.15	6.89 ± 0.08	90.38 ± 1.78	6.96 ± 0.06
WT_Testo	9.0 ± 0.22^*^	7.14 ± 0.08	52.05 ± 2.67^*^	6.34 ± 0.15
WT_MCC950	6.3 ± 0.13	6.9± 0.07	88.76 ± 3.06	7.41 ± 2.15
WT_MCC950+Testo	6.4 ± 0.22^#^	7.17 ± 0.10	78.38 ± 3.0^*^	6.89 ± 0.12

Data represent the mean ± S.E.M (n = 3–10 mice per group). Two-way ANOVA:

* p < 0.05 vs. WT_Vehicle;

# *p < 0.05 vs. WT_Testo. Emax, maximal response; pEC_50_, negative logarithm of the EC_50_; PE, phenylephrine; ACh, acetylcholine; Testo, testosterone; MCC950, NLRP3 antagonist; WT, wild type*.

To determine whether AR mediate testosterone-induced vascular dysfunction, Flu, an AR antagonist, was used. Flu prevented testosterone-induced increased contractile responses to PE (Figure 3C and Table 5) and the impaired ACh vasodilation (Figure 3D and Table 5). No differences were observed between vascular reactivity of thoracic aortic rings of WT mice incubated with Flu and vehicle.

**Table 5:** Maximal response and pEC_50_ values for PE-induced contraction and ACh-induced relaxation in aortas incubated with Testo, Flu, Flu+Testo or Vehicle from WT mice.

	**PE**		**Ach**	
	**Emax (mN)**	**pEC_**50**_**	**Emax (%)**	**pEC_**50**_**
WT_Vehicle	5.82 ± 0.16	6.83 ± 0.08	85.0 ± 2.2	7.15 ± 0.09
WT_Testo	8.63 ± 0.21^*^	7.1 ± 0.08	53.1 ± 2.67^*^	6.32 ± 0.14^*^
WT_Flu	5.44 ± 0.12	7.01 ± 0.07	83.76 ± 2.4	7.38 ± 0.12
WT_Flu+Testo	5.32 ± 0.23^#^	6.78 ± 0.12	86.72 ± 2.5^#^	7.16 ± 0.09^#^

Data represent the mean ± S.E.M (n = 3–10 mice per group). Two-way ANOVA:

* p < 0.05 vs. WT_Vehicle;

# *p < 0.05 vs. WT_Testo. Emax, maximal response; pEC_50_, negative logarithm of the EC_50_; PE, phenylephrine; ACh, acetylcholine; Testo, testosterone; Flu, Flutamide, androgen receptor antagonist; WT, wild type*.

### Supraphysiological Testosterone Levels Induce Vascular Generation of Mitochondria-Derived Reactive Oxygen Species

**Figure 4:**
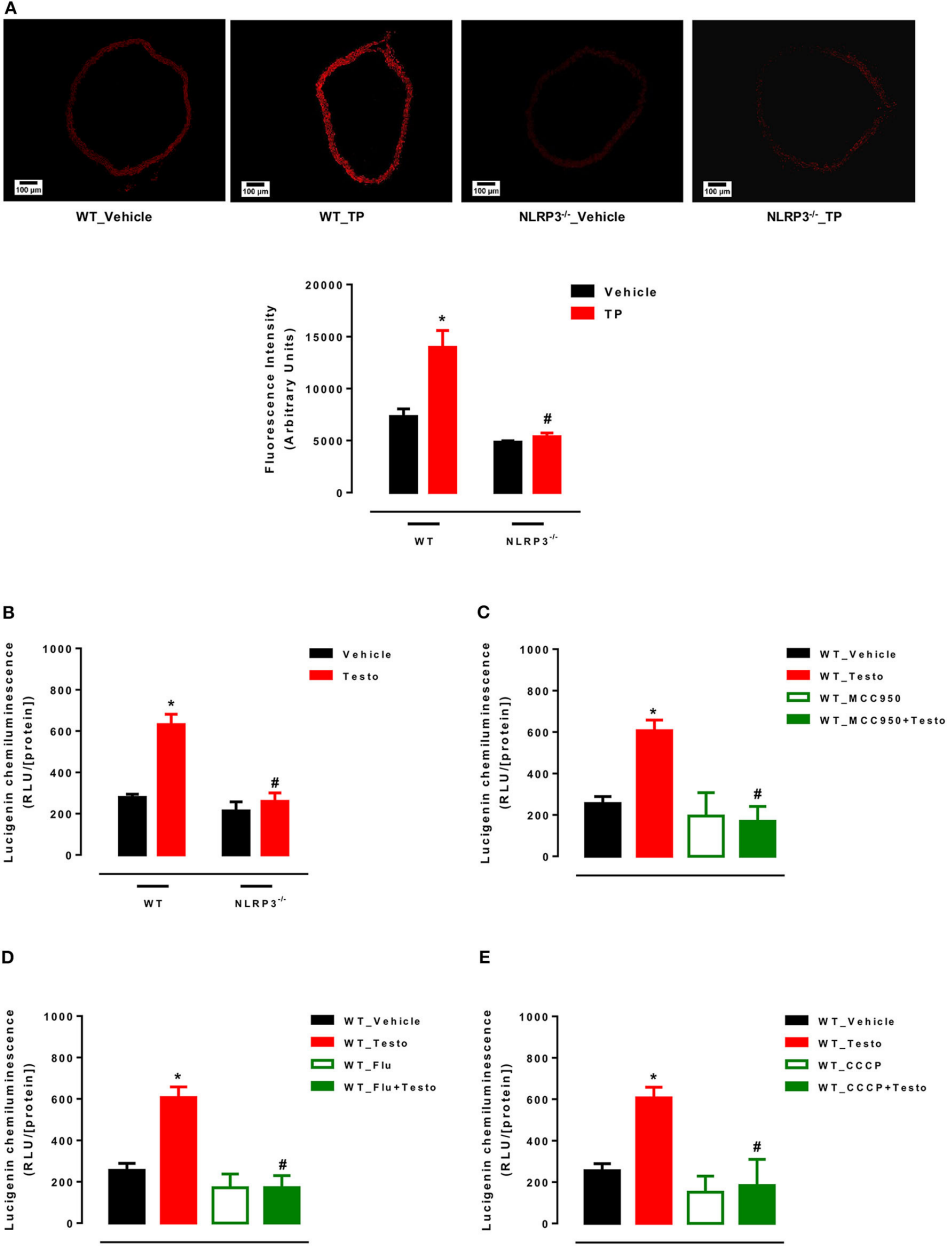
Supraphysiological testosterone levels induce vascular generation of reactive oxygen species. Vascular ROS generation was measured by dihydroethidine in aortas from WT_Vehicle, WT_TP, NLRP3^−/−^_Vehicle and NLRP3^−/−^_TP mice **(A)**; superoxide anion generation – measured by lucigenin in vessels incubated with Testo or vehicle (WT_Vehicle, WT_Testo, NLRP3^−/−^_Vehicle and NLRP3^−/−^_Testo) **(B)**; effects of MCC950 **(C)**, Flu **(D)** and CCCP **(E)** on testosterone effects in aortas isolated from WT mice and incubated with vehicle or testosterone. Data are expressed as mean ± SEM (*n* = 5). *p < 0.05 vs. WT_Vehicle; ^#^*p* < 0.05 vs. WT_TP; ^#^*p* < 0.05 vs. WT_Testo.

Initially, ROS generation in thoracic aortas of WT and NLRP3^−/−^ mice treated with TP or vehicle was determined. TP treatment increased ROS vascular generation in WT mice, but not in NLRP3^−/−^ mice (Figure 4A).

Mitochondrial ROS generation was also determined in thoracic aortas of WT and NLRP3^−/−^ mice incubated with testosterone or vehicle. Testosterone increased mROS in aortas from WT mice, but not in NLRP3^−/−^ aortas (Figure 4B). The contribution of NLRP3 inflammasome (Figure 4C), AR (Figure 4D) and mitochondrial electron transport chain (Figure 4E) to ROS generation was also determined *in vitro* in aortas from WT mice. Mitochondrial uncoupling by CCCP, an inhibitor of oxidative phosphorylation, NLRP3 deletion and blockade of AR abrogated testosterone-induced vascular ROS generation.

**Figure 5:**
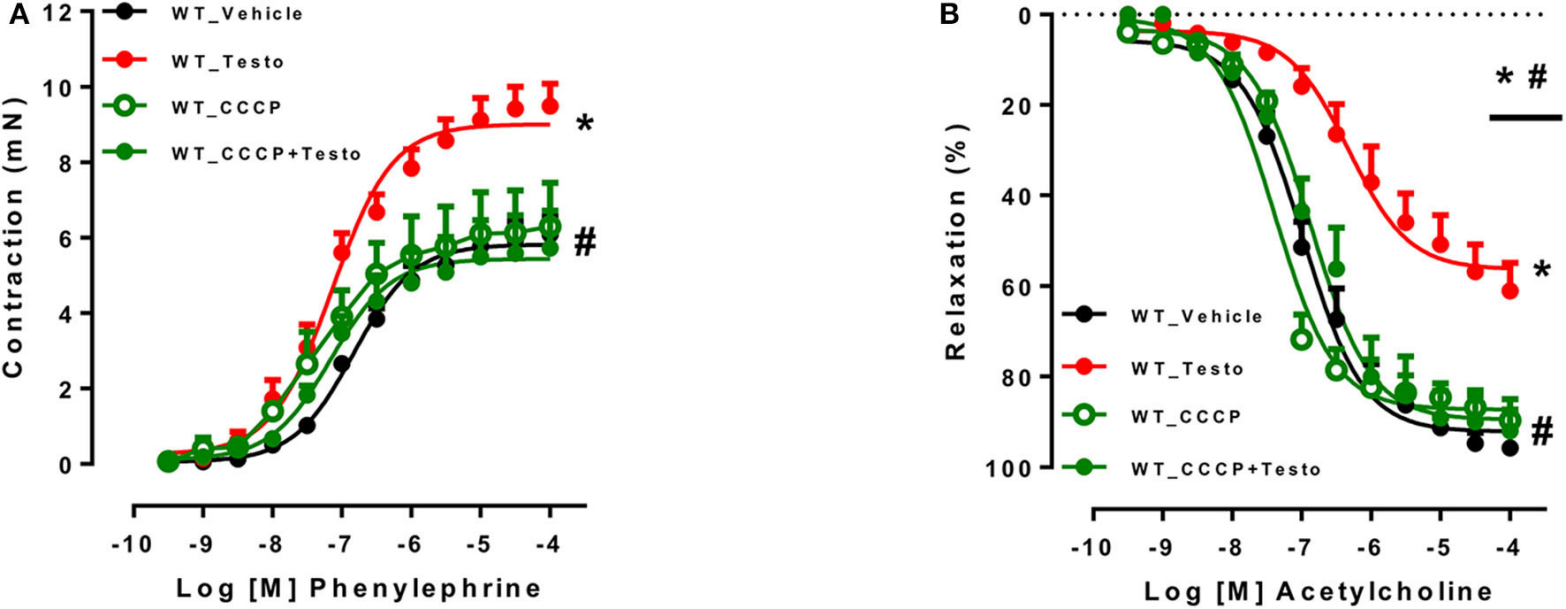
Testosterone induces vascular dysfunction via the generation of mitochondrial reactive oxygen species. Concentration-response curves to phenylephrine - PE **(A)** and acetylcholine - ACh **(B)** were performed in aortic rings incubated with testosterone (10^−6^ M for 2 h) or vehicle isolated from WT mice. The effects of CCCP (10^−6^ M for 30 min) on testosterone effects were determined. Data are expressed as mean ± SEM (*n* = 3–10). *p < 0.05 vs. WT_Vehicle; ^#^*p* < 0.05 vs. WT_Testo.

To determine whether mitochondrial ROS contribute to the vascular effects of testosterone, experiments were performed in the presence of the mitochondrial uncoupler CCCP. CCCP prevented testosterone-induced increased in contractile responses to PE (Figure 5A and Table 6) and the impaired ACh vasodilation (Figure 5B and Table 6). No differences were observed between vascular reactivity of thoracic aortic rings of WT mice treated with CCCP and vehicle.

**Table 6:** Maximal response and pEC_50_ values of PE-induced contraction and ACh-induced relaxation in aortas incubated with Testo, CCCP, CCCP+Testo or Vehicle from WT mice.

	**PE**		**Ach**	
	**Emax (mN)**	**pEC_**50**_**	**Emax (%)**	**pEC_**50**_**
WT_Vehicle	5.82 ± 0.16	6.83 ± 0.08	92.14 ± 1.7	6.99 ± 0,06
WT_Testo	9.0 ± 0.22^*^	7.14 ± 0.08	56.3 ± 2.7^*^	6.32 ± 0.13^*^
WT_CCCP	6.03 ± 0.33	7.28 ± 0.22	87.3 ± 2.2	7.4 ± 0.11
WT_CCCP+Testo	5.44 ± 0.3^#^	7.16 ± 0.18	89.5 ± 2.8^#^	6.88 ± 0.10^#^

Data represent the mean ± S.E.M (n = 3–10 mice per group). Two-way ANOVA:

* p < 0.05 vs. WT_Vehicle;

# *p < 0.05 vs. WT_Testo. Emax, maximal response; pEC_50_, negative logarithm of the EC_50_; PE, phenylephrine; ACh, acetylcholine; Testo, testosterone; CCCP, carbonyl cyanide m-chlorophenyl hydrazone, inhibitor of oxidative phosphorylation; WT, wild type*.

## Discussion

The primary goal of the present study was to uncover whether supraphysiological levels of testosterone induce NLRP3 inflammasome activation and consequent vascular dysfunction. *In vitro* studies using thoracic aortas and *in vivo* treatment of WT and NLRP3^−/−^ mice demonstrated that supraphysiological levels of testosterone activate the NLRP3 inflammasome in vascular cells and cause vascular dysfunction. Our study provides *in vivo* evidence that NLRP3 inflammasome activation mediates proinflammatory effects of elevated levels of testosterone, thereby contributing to vascular dysfunction. Antagonism of testosterone receptors and inhibition of mitochondrial ROS generation prevent testosterone-induced vascular dysfunction *in vitro*. These findings, for the first time, demonstrate a key role for both increased ROS-mediated signaling and activation of NLRP3 inflammasome to vascular damage induced by testosterone.

Whereas, the consequences of testosterone deprivation in the cardiovascular system have been investigated (44), the effects of supraphysiological levels of the hormone have been surprisingly little studied. Evidence indicates that supraphysiological levels of testosterone affect the function and structure of the cardiovascular system. Sprague-Dawley rats treated with supraphysiological doses of testosterone show eccentric cardiac hypertrophy mediated by ERK 1/2 and mTOR phosphorylation (45). Increased systemic testosterone also decreases relaxation of human pulmonary arteries and veins (46).

In the present study, we addressed direct and long-term effects of testosterone on NLRP3 inflammasome-mediated vascular dysfunction. The innate and adaptive immune responses significantly influence both acute and chronic changes in cardiovascular phenotype that lead to clinical cardiovascular abnormalities (47–50).

Members of the NLR family have emerged as important sensors involved in the immune responses to pathogens and inflammatory diseases. NLRP3, a well-characterized member of the NLR family, regulates the assembly of the inflammasome, a multimeric complex protein that activates inflammatory caspase-1, which cleaves pro-interleukin-1β (pro-IL-1β) and pro-IL-18 into their mature and biologically active forms (51). Vascular cells can detect and respond to a variety of signals that are indicative of cell damage, including environmental irritants, endogenous danger signals, pathogens, and mitochondria-derived ROS, leading to the release of cytokines, chemokines and hormones (52, 53). The present study supports that the NLRP3 inflammasome is involved in the vascular dysfunction triggered by high testosterone levels. First, NLRP3 gene deletion prevented testosterone-induced hypercontractility of vascular smooth muscle (VSM) and endothelial dysfunction both *in vitro* and *in vivo*. Vascular alterations in response to testosterone *in vitro* were also partially prevented in the presence of MCC950, further indicating that NLRP3 inflammasome is key to vascular damage induced by testosterone. The observations that both chronic treatment of mice with testosterone propionate and incubation of aortas with testosterone increase vascular caspase-1 expression as well as IL-1β levels provide further support to the idea that testosterone induces vascular NLRP3 inflammasome activation.

Our group recently demonstrated that NLRP3 receptor inhibition restores the functional integrity of resistance mesenteric arteries of diabetic animals (38). Previous studies also showed that IL-1β, a key immunoregulatory and proinflammatory cytokine produced by the inflammasome, reduces endothelium-dependent relaxation, increases vascular contractility, as well as VSM cells migration and proliferation with consequent vascular remodeling (37, 54–56).

ROS production, especially from the mitochondria, triggers NLRP3 inflammasome activation (57). Testosterone induces long-term ROS production by genomic mechanisms in a time- and concentration-dependent manner. Also, it stimulates short-term ROS production by unique nongenomic mechanisms in VSM cells from hypertensive animals (40). In that the present study *in vitro* treatment of vascular segments with testosterone increased vascular ROS generation. Interestingly, chronic treatment with TP and incubation of arteries with testosterone increased IL-1β, the end product of NLRP3 inflammasome activation (58). Finally, the findings that vascular ROS generation in response to testosterone incubation is prevented by MCC950, Flu and CCCP, imply AR and mitochondria on testosterone-induced ROS generation in VSM cells (41).

To determine NLRP3 inflammasome activation in testosterone-induced ROS generation and vascular dysfunction *in vivo*, we used NLRP3^−/−^ mice treated with TP. Our analysis revealed that inflammasome signaling is critical to testosterone-induced vascular dysfunction. Lack of NLRP3 inflammasome abolished testosterone-induced ROS generation and IL-1β production, indicating an interplay between ROS and NLRP3, as trigger and effector molecules.

The role of mitochondrial ROS in the vascular effects of high testosterone concentrations were further investigated in our study. CCCP prevented the deleterious effects of testosterone on vascular function. This is consistent with earlier data from our group, showing that mitochondrial ROS from the perivascular adipose tissue (PVAT) of obese mice induces vascular dysfunction (59).

AR modulate inflammatory processes and activation of components of the immune system. Accordingly, male mice lacking AR in monocytes/macrophages exhibit less eosinophil recruitment and lung inflammation due to impaired M2 polarization (60). On the other hand, animals with deletion of AR in macrophages/monocytes are protected from atherosclerosis (61) and depletion of AR has protective effects on abdominal aortic aneurysm development in WT mice (62). In this study Flu prevented testosterone effects on vascular function and ROS generation, supporting that AR contribute to testosterone-induced activation of inflammasome and oxidative stress. In addition to inhibition of mitochondrial ROS generation and NLRP3 inflammasome activation, blockade of AR prevented impaired vascular contractile and relaxant responses. The evidence that testosterone, via AR, promotes calcium (Ca^2+^) influx (63) and that intracellular Ca^2+^ is crucial for the generation of mitochondrial ROS (64) is consistent with our hypothesis that supraphysiological levels of testosterone induces vascular dysfunction via increased mitochondrial ROS-mediated activation of NLRP3 inflammasome. Since testosterone activates other receptors, including GPRC6A and GPER (65), further studies are warranted for a more detailed investigation on the relative contribution of AR to the vascular complications induced by supraphysiological concentrations of testosterone. Meanwhile, our study highlights classical AR in the pathogenesis of vascular dysfunction.

The current study has strengths and limitations. The experiments rigorously followed stringent quality criteria in experimental research such as randomization, manipulation and evaluations performed in a blinded fashion, controlled physiological parameters and use of control groups of health animals. A major limitation of the current study was the *in vivo* approach, which restricts mechanistic investigations of tissue-specific inflammatory pathways, since it does not allow identification of the specific cell types responsible for the observed systemic changes. Considering that the primary goal of this study was to directly investigate mechanisms of impaired vascular dysfunction induced by high systemic levels of testosterone, to overcome this limitation we also addressed the effects of testosterone on isolated vessels. The analysis of molecular mechanisms involved in testosterone-induced activation of NLRP3 was supported by the *in vitro* approaches. However, results observed on *in vitro* assays, although important, often fail to translate to similar results *in vivo*. Additionally, although DHE has been extensively used DHE to detect ${\text{O}}_{2}^{-}$ in cells or systems, the interference from other oxidative radicals prevents a fine tune quantification of ${\text{O}}_{2}^{-}$ (66, 67). Since every method has shortcomings, we used a combination of DHE, lucigenin and pharmacological inhibitors to better evaluate ${\text{O}}_{2}^{-}$ production. In the present study we have not used aromatase or 5-alpha reductase inhibitors to determine whether estradiol or dihydrotestosterone contribute to testosterone effects. However, in previous studies, testosterone effects were not blocked or modified by anastrozole (aromatase inhibitor). Finally, the time of treatment as well as the dose of testosterone were based on previous studies that investigated other metabolic and cardiovascular parameters in different experimental models.

## Conclusion

Our findings provide evidence that supraphysiological levels of testosterone impairs vascular function via activation of the NLRP3 inflammasome in vascular cells. The generation of mitochondrial ROS is crucial for activation of the NLRP3 inflammasome. Pharmacologic inhibition or genetic deletion of the NLRP3 in mice protects from testosterone-induced vascular dysfunction. Our study highlights the importance of NLRP3 inflammasome in vascular dysfunction promoted by supraphysiological levels of testosterone.

## Data Availability Statement

The datasets generated for this study are available on request to the corresponding author.

## Ethics Statement

The animal study was reviewed and approved by Ethics Committee on Animal Use (CEUA) of the University of São Paulo, Ribeirao Preto, Brazil (Protocol n° 032/2018).

## Author Contributions

JA, RC, NL, and RT designed the study. JA, RC, CP, AF, and CS conducted the experiments. JA, RC, and RT provided supplies and analytical tools. JA, RC, CP, and AF performed the data analysis. JA, RC, FC, NL, and RT wrote the paper. All authors contributed to the article and approved the submitted version.

## Conflict of Interest

The authors declare that the research was conducted in the absence of any commercial or financial relationships that could be construed as a potential conflict of interest.

## Abbreviations

ACh: acetylcholine
AR: androgen receptor
BKCa: large-conductance voltage- and calcium-activated potassium channels
BSA: bovine serum albumin
CCCP: carbonyl cyanide m-chlorophenyl hydrazone
CONCEA: National Council for Animal Experimentation Control
DHE: dihydroethidine
Emax: maximum response
Flu: flutamide
IL-12: interleukin-12
IL-1β: interleukin-1β
KCa: calcium-activated potassium channels
KCl: potassium chloride
Kv: voltage-sensitive potassium channels
MCC950: NLRP3 receptor inhibitor
mROS: mitochondrial reactive oxygen species
NADH: adenine nicotinamide dinucleotide
NLRP3^−/−^: NLRP3 knockout mouse
NLRs: NOD-like receptors
NO: nitric oxide
NOD: nucleotide-binding oligomerization domain-like receptors
${\text{O}}_{2}^{-}$: superoxide anion
PE: phenylephrine
pEC_50_: negative logarithm of the EC_50_
pro-IL-18: pro-interleukin-18
pro-IL-1β: pro-interlukin-1β
PRR: pattern recognition receptors
PVAT: perivascular adipose tissue
RLU: relative luminescence units
ROS: reactive oxygen species
SEM: standard error of the mean
SKCa: small-conductance voltage- and calcium-activated potassium channels
SNP: sodium nitroprusside
TBS: Tris-buffered saline
Testo: testosterone
TNFα: tumor necrosis factor alpha
TP: testosterone propionate
Vehicle: peanut oil
VSM: vascular smooth muscle
WT: wild type.
